# Isolation and characterization of non tuberculous mycobacteria from humans and animals in Namwala District of Zambia

**DOI:** 10.1186/1756-0500-7-622

**Published:** 2014-09-09

**Authors:** Sydney Malama, Musso Munyeme, Sydney Mwanza, John Bwalya Muma

**Affiliations:** Institute of Economic and Social Research, University of Zambia, P. O. Box 30900, Lusaka, Zambia; Biomedical Sciences Department, Tropical Diseases Research Centre, P. O. Box 71769, Ndola, Zambia; Department of Disease Control, University of Zambia, P. O. Box 32379, Lusaka, Zambia

**Keywords:** Namwala, Non tuberculous mycobacteria, Zambia

## Abstract

**Background:**

The genus *Mycobacterium* contains more than 100 species, most of which are classified as non-tuberculous mycobacteria (NTM). In Zambia, the NTM are slowly becoming recognized as pathogens of major public health significance with the advent of Human Immunodeficiency Virus (HIV) and Acquired Immunodeficiency Syndrome (AIDS). This study aimed at reporting the isolated NTM and ascertains their zoonotic potential and diagnostic significance in Zambia.

**Method:**

A total of 100 sputum samples were collected from three health facilities from suspected pulmonary tuberculosis human patients. In addition, 67 lymph node tissue samples from cattle and 14 from Kafue lechwe (*Kobus leche kafuensis*) showing tuberculosis-like lesions were collected. The samples were appropriately decontaminated and cultured on Middlebrook 7H10 and Stone brink. The isolates were then identified accordingly using the 16S ribosomal RNA analysis method.

**Results:**

A total of 8 NTM were isolated from human sputum, 12 from cattle and 1 from the Kafue lechwe. The identified NTM included *M. intracellulae*, *M. abscess*, *M. chimaera*, *M. bolleti*, *M. fortuitum* and *M. stomatopae* sp. Nov.

**Conclusion:**

The isolation of NTM from humans and animals at the interface in Namwala district has highlighted the clinical significance and diagnostic challenge. The epidemiological investigation of NTM in the study area is therefore recommended. This should include sampling from environmental sources such as water and soil.

## Background

The genus *Mycobacterium* contains more than 100 species, most of which are classified as non-tuberculous mycobacteria (NTM) [1] and mycobacteria belonging to the *Mycobacterium tuberculosis* complex (MTC) [2].

The genus *Mycobacterium*, closely related by its cell wall antigens to the genera *Corynebacterium* and *Nocardia*, is presently classified in the order Actinomycetales and family Mycobacteriaceae [3]. Mycobacteria are aerobic, non-motile, non-spore forming, Gram positive, straight or slightly curved rods 1.5 to 4 μm long and 0.3 to 0.5 μm wide. Their cell wall has a high lipid content which once stained with carbol fuchsin cannot be decolorized by acid alcohol thus their name ‘acid fast bacteria’ [4].

Non-tuberculous mycobacteria encompass all mycobacteria other than the species belonging to the MTC and *Mycobacterium leprae*. Many NTM are saprophytic environmental species, and some may cause disease in animals and humans [5]. The *Mycobacterium avium* complex (MAC) is the most commonly encountered group of NTM. The clinically most important members of MAC are *M. intracellulae* and *M. avium* [6]. Other NTM species include *M. chelonae*, *M. kansasii*, *M. marinum and M. fortuitum* [1]. In Zambia, the NTM are slowly becoming recognized as pathogens of major public health significance with the advent of Human Immunodeficiency Virus (HIV) and Acquired Immunodeficiency Syndrome (AIDS) [7, 8].

Diagnosis of mycobacteria in Zambia is mainly by microscopy [9]. However, the microscopic method does not differentiate species belonging to the mycobacterium genus. Therefore, sequencing of the 16S ribosomal RNA genes is by far the most preferred molecular tool for phylogenetic and taxonomic studies and is suitable for identification of bacteria [10].

This study aimed at reporting, the clinical and diagnostic significance of the isolated NTM in Zambia.

## Methods

### Study area

The study was conducted in Namwala district, which is one of the districts with the highest number of cattle farmers in Zambia. The district, which is situated in the Southern Province of the country, also has the highest known TB prevalence in both animals (domestic and wild) and human [11, 12]. Approximately a quarter of its traditional land is covered by the flood plains of the Kafue River. It covers an estimated total area of about 10,000 square kilometres and lies between latitudes 15 and 17°S of the equator and longitude 25 and 27°E. Namwala district is located within the Kafue basin, which is one of the lacustrine wetlands supporting close to 300,000 herds of cattle and 44,000 Kafue lechwe [13]. Human population in the district is estimated at 102,000 people according to the Zambia’s 2010 census of population and housing [14].

### Study design and sampling

The study was designed as a cross section study sampling from both animals and humans in the area was done between April 2011 and July 2012. For human sampling, suspected TB patients seeking medical attention at three local health centres in the districts, namely the Central Namwala District Hospital, the Maala Rural Health Centre, and the Chitoongo Rural Health Centre comprised the target population for the human study. The suspected pulmonary TB patients were patients who had a cough of more than two weeks; they also reported loss of appetite and night sweats. For the cattle study, the target population comprised cattle carcasses showing gross tuberculous-like lesions at meat inspection at one of the two abattoirs in the Namwala district during the study periods. Our sampling therefore was purposive, where subjects were selected based on the above described characteristics in order to increase the chances of isolating mycobacteria. The cattle that were slaughtered at the abattoir were drawn from the villages where patients seeking medical attention at the three health facilities came from. For Kafue lechwe, lymph node tissue samples from historical specimen collected in 2011 under a special research licence were analysed. Ecological details of the sampling sites are described in the earlier report by Muma et al. [15]. In cattle and Kafue lechwe sampling was done by Veterinary surgeons.

### Humans

A total of 150 TB patients were screened for tuberculosis, but only 110 subjects submitted sputum samples. Informed written consent in English or the local language (*Tonga*) was provided by the patients. From the submitted sputum samples, 10 were discarded because of poor quality. Therefore, only 100 sputum samples from pulmonary TB patients were processed.

### Cattle

A total of 288 cattle slaughtered at one of the two abattoirs located in the study area were examined for gross lesions according to the standard post-mortem abattoir examination procedures [16]. The specimens from animals were collected as part of the routine abattoir work. Sixty-seven lymph node tissues, exhibiting gross lesions suggestive of BTB were collected for further analysis during the study period.

### Kafue lechwe

A set of 14 lymph node tissue samples with lesions suggestive of BTB, collected from 14 Kafue lechwe hunted under a special research license from Zambia Wildlife Authority, were processed (ZAWA).

### Ethical approval

The study was approved by IRB - ERES converge ethical review committee, Lusaka, Zambia (Ref: 2012-Mar-001) and permission to perform the study in the area was obtained from the District Medical Officer (DMO) and the Provincial Veterinary officer (PVO). Wildlife samples were collected under a special research license from Zambia Wildlife Authority (ZAWA).

### Laboratory analysis

#### Sputum samples

##### ZN staining

Sputum was first smeared on a glass slide and fixation was conducted by heating 65°C to 75°C) in a Class 1 exhaust protective cabinet until the smeared material was dried and fixed to the glass slide. This was then flooded with strong carbol fuschin, and heated gently for 3–5 minutes. This was followed by adequate rinsing with water and decolourised for 2–3 min with a (3% v/v) acid-alcohol solution, another water rinse and then replaced with fresh acid-alcohol for 3–4 minutes until the slide remained a faint pink colour. It was then rinsed well with water and counter stained with (1% w/v) methylene blue for 30 seconds before rinsing again with water and allowing drying. It was visualized under bright field microscopy (manufactured by Olympus, USA) using an immersion oil and using the X 10 magnification for focusing and the X 100 magnification for reading. About one hundred fields were examined for every slide. Mycobacteria are “acid-fast bacilli” (AFB) and are seen as red rods under this stain [17].

##### Culture

After the ZN staining, sputum smear positive samples were stored in cetylpyridium chloride transport media (Difco, Detroit, MI, USA) and kept at ambient temperature at the Namwala district hospital until they were taken to the Chest Disease Laboratory in Lusaka. Samples were decontaminated by mixing in 4% sodium hydroxide (NaOH) with an equal volume of sputum sample, mixed and placed in a water bath (37°C) for 1 hour with intermittent shaking. This was followed by centrifugation at 3500 *g* for 30 minutes. The supernatant was discarded and the pellet was re-suspended in 1 ml of 1Χ phosphate buffered saline (PBS). Each sample was cultured on Stonebrink and Middlebrook 7H10 media (BD diagnostics®) and incubated at 37°C. The cultures were examined for growth twice weekly for 8 weeks, after which definitive results were obtained and recorded. Successfully grown cultures were transported to the University of Zambia, School of Veterinary Medicine for storage and further analysis.

#### Tissue samples –cattle and lechwe

##### Culture

All the 67 samples from cattle and 14 from Kafue lechwe were processed according to standard procedures described by Grange et al. [17]. The decontaminated homogenates were inoculated on Stonebrink (which contains pyruvate) (BD Diagnostics, MD) and Middlebrook 7H10 (BD Diagnostics, MD) slants and incubated at 37°C for 8 weeks with weekly observation.

##### ZN staining

Grown cells from cultured tissue homogenates were smeared on glass and were processed like sputum above. Mycobacteria are “acid-fast bacilli” (AFB) and were seen as red rods under this stain [17].

All grown cultures with typical mycobacterial morphology and were acid fast by the ZN staining method were stored −18°C for further processing in Oslo, Norway.

#### DNA extraction and purification

Mycobacterial colonies from secondary grown cultures on solid media were harvested as a loop full of colony material, suspended in 200 μl ultrapure molecular grade water (MQ) and heated at 95°C for 20 minutes. The crude lysates were purified using the NucliSENS easyMAG automated machine (Biomerieux, Netherlands) following the manufacturer’s protocol. The purified DNA was stored at −20°C for further analysis in Oslo, Norway.

#### 16 S ribosomal RNA analyses

The purified Genomic DNA was characterized based on sequencing of the 16S ribosomal RNA gene using the following primers: 16S8F (AGAGTTTGATCMTGGYTCAG) and 16SM259 (TTTC ACGAACAACGCGACAA) [18]. The obtained sequences were edited and analysed in the bioinformatics software Bio-edit (http://www.mbio.%20ncsu.edu/Bio%20Edit/bioedit.html) and the sequences were blasted at the NCBI Blast database (National Centre for Biotechnology Information). The species identification was strictly determined based on the maximum score and maximum identity values of the reference sequences in NCBI Blast alignment. Isolates with the maximum scores and maximum identities of 100 or 99% and documented as approved nomenclature were accepted for species identification [5, 19].

## Results

In this paper we only report the NTMs isolated from humans, cattle and Kafue lechwe. The *Mycobacterium tuberculosis* complex has been reported elsewhere [20–22].

### Human

Out of the sputum samples obtained from 100 humans, acid fast bacilli were detected in direct smears from 65 patients. Mycobacteria were detected by culture in 55 of these. Based on 16 S ribosomal RNA analysis 9 isolates were identified as NTMs (Table 1).

**Table 1:** **Non tuberculous mycobacteria isolated from humans**, **livestock and** -**wildlife in Namwala district**, **Zambia**

Host	Specie	Frequency (n)
Human	*M. intracellulae*	4
	*M. abscess*	2
	*M. chimaera*	2
	*M. bolleti*	1
Cattle	*M. fortuitum*	4
	*M. intracellulae*	8
Kafue lechwe	*M. stomtopae sp. nov*	1

The identification was based on the homology values of <98.7 according to [19].

### Cattle

Out of the tissue samples obtained from 67 cattle carcasses, acid fast bacilli were detected by direct smear in 55 animals. Mycobacteria were detected by culture in 47 samples. Based on 16 S ribosomal RNA analysis 12 isolates were identified as NTMs.

### Kafue lechwe

Acid fast bacilli were detected in direct smear in 27 of the 39 samples and mycobacteria were detected by culture in 8 of the samples. Based on 16S ribosomal RNA analysis only one isolate was identified as an NTM (Table 1).

## Discussion

In the present study, a range of NTMs from human and animal in Namwala district, an agro-pastoral district located in Southern province of Zambia were detected. Genotypic classification involving sequencing of the 16S ribosomal RNA genes is regarded as the definitive standard for determining phylogenic relationship of bacteria. The homology values have a profound effect on interpreting this relationship. Homology values of <98.7 (97%) according to Austin, 2011 [19] indicates membership of different species. One NTM was isolated from the Kafue lechwe and it was identified as *Mycobacterium stomatepiae sp nov*, This bacteria is associated with causing diseases in Fish [19]. The Kafue lechwe is a semi aquatic animal, grazing in water levels of about 50 cm, and its therefore most likely that the isolated *M. stomatepiae* sp nov., infection was contracted from water or soil in the Kafue River possibly contaminated by infected fish [23].

This study further isolated and identified NTM from both humans and cattle. The common NTM isolated was the *Mycobacterium intracellulae*. This NTM has been isolated from humans, cattle and wildlife elsewhere [5, 6, 18, 24]. NTM are otherwise referred to as environmental bacteria, hence exposure to both contaminated soils and water could be a source of infection to humans and animals. Further, other studies have suggested that consumption of unpasteurized milk could also be a source of NTM for humans suggesting a zoonotic potential of these bacteria [25]. In the same study, *M. bovis* with similar genotypes was isolated from some of the cattle and humans. This however, suggested the zoonotic potential of *M. bovis* in the study area [21]. In Zambia however, NTM have been isolated from chronically ill patients most of whom were suffering from HIV infection [8, 24].

The isolation of NTM from human sputum and granulomatous lesions of affected animals raises the question of these bacteria in the observed disease pathology. Based on the present data, it seems certain that the cause of the observed disease in some of the patients and animals was due to the isolated NTM. This is so because NTM and MTC were not simultaneously isolated in all the species under study – humans and animals.

This study further shows that in cases where potentially pathogenic NTM are isolated from mycobacteria cultures of tuberculosis –like lesions and human sputum, the non –use of additional selective culture techniques could lead to misinterpretations of the diagnostic test results. This is in view of the fact that tuberculosis like lesions in wildlife and livestock is highly associated with *Mycobacterium bovis* [26] and human tuberculosis is also associated with *Mycobacterium tuberculosis* [27]. This hypothesis is supported by a study done in Chad which suggested that 72% of the tuberculosis lesions in cattle carcasses detected through standard meat inspection were caused by pathogens other than *M. bovis* [28].

## Conclusion

This study has isolated NTM from humans and animals in Namwala district. It has therefore, highlighted the contribution of NTM in disease pathology and diagnostic challenges. We recommended epidemiological investigation of NTM and the use of appropriate diagnostic tools in the study area which should include sampling from environmental sources such as water and soil.
